# Optimization of Nitrogen Rate and Planting Density for Improving Yield, Nitrogen Use Efficiency, and Lodging Resistance in Oilseed Rape

**DOI:** 10.3389/fpls.2017.00532

**Published:** 2017-05-09

**Authors:** Shahbaz Khan, Sumera Anwar, Jie Kuai, Sana Ullah, Shah Fahad, Guangsheng Zhou

**Affiliations:** College of Plant Science and Technology, Huazhong Agricultural UniversityWuhan, China

**Keywords:** rapeseed, plant density, yield, lodging, nitrogen use efficiency

## Abstract

Yield and lodging related traits are essential for improving rapeseed production. The objective of the present study was to investigate the influence of plant density (D) and nitrogen (N) rates on morphological and physiological traits related to yield and lodging in rapeseed. We evaluated Huayouza 9 for two consecutive growing seasons (2014–2016) under three plant densities (LD, 10 plants m^−2^; MD, 30 plants m^−2^; HD, 60 plants m^−2^) and four N rates (0, 60, 120, and 180 kg ha^−1^). Experiment was laid out in split plot design using density as a main factor and N as sub-plot factor with three replications each. Seed yield was increased by increasing density and N rate, reaching a peak at HD with 180 kg N ha^−1^. The effect of N rate was consistently positive in increasing the plant height, pod area index, 1,000 seed weight, shoot and root dry weights, and root neck diameter, reaching a peak at 180 kg N ha^−1^. Plant height was decreased by increasing D, whereas the maximum radiation interception (~80%) and net photosynthetic rate were recorded at MD at highest N. Lodging resistance and nitrogen use efficiency significantly increased with increasing D from 10 to 30 plants m^−2^, and N rate up to 120 kg ha^−1^, further increase of D and N decreased lodging resistance and NUE. Hence, our study implies that planting density 30 plants m^−2^ can improve yield, nitrogen use efficiency, and enhance lodging resistance by improving crop canopy.

## Introduction

Rapeseed (*Brassica napus* L.) is the third most important worldwide oil crop, after soybean and palm oil. In 2014, rapeseed was produced on 36.12 M ha globally, mainly in China (7.59 M ha), Canada (8.07 M ha), and the European Union (6.71 M ha; Food and Agriculture Organization of the United Nations, 2017). It is one of the major oilseed crops in China, yielding 11.6 million tons per year. Yangtze River Basin is the largest rapeseed production region in China, with about 90% of national rapeseed production (Li et al., 2016). Many climatic and agronomic factors affect rapeseed yield, including lodging, plant density and fertilizers rates. Direct drill planting and mechanized harvesting has enabled farmers to gain higher yield of rapeseed from attaining higher plant densities (Hu et al., 2016). Although previous studies reported that after a saturation threshold, yield per unit area does not increase with plant density because of high intra specific competition for resources (Raey and Ghassemi-Golezani, 2009). Al-Barzinjy et al. (1999) showed that dry biomass per plant, seed weight per plant and number of pods per plant decrease with increasing plant density, whereas seed yield m^−2^ peaks at 50 plants m^−2^ and decreases at higher densities, following a parabolic curve relationship. Likewise, relatively low plant densities decrease the overall rapeseed yield by promoting vegetative but not reproductive growth (Bagheri et al., 2011). Therefore, desired plant density needs to be attained before the onset of the reproductive stage, a stage at which canopy reaches its maximum size, by changing seeding rates and row spacing (Purcell et al., 2002; Wang C. et al., 2015; Wang R. et al., 2015).

Nitrogen (N) is a macronutrient that significantly affects yield and growth in rapeseed plants. However, plant requirements for N vary with cultivar, growth stage of plant, N utilization efficiency, soil type, climate, and type of N application (Sidlauskas and Bernotas, 2003; Berry et al., 2010). Previous studies aimed to optimize the rate of N to increase rapeseed yield (Kazemeini et al., 2010). Applying fall nitrogen to oilseed rape is common practice in China and Europe. In the last few years, farmers have changed the crop rotation; in consequence, the sowing date is often delayed. In addition, they increasingly pass on plowing during seedbed preparation to reduce energy costs. In order to compensate for delayed sowing, they additionally apply about 30–50 kg N/ha in fall, often directly upon the stubble of the preceding crop, to ensure adequate crop growth before winter. Application of N increases the yield by improving growth, but its excessive use leads to higher production costs, increase risk of nitrate leaching and water contamination and reduce nitrogen use efficiency (Sieling and Kage, 2010; Zhang et al., 2013). Additionally, high N rates promote lodging by increasing plant height and raising the center of gravity and by decreasing lignin and cellulose content as well as stem diameter and cell wall thickness of basal internodes (Wang et al., 2012; Zhang et al., 2013). Despite of its capacity to absorb N from the soil in fall and spring, the N use efficiency of rapeseed is low which is around half that for cereals (Sylvester-Bradley and Kindred, 2009). Improving the N use efficiency of rapeseed is therefore very important to ensure the competitiveness of this crop at agronomic, environmental, and economic levels. Improving yield under low N levels is a key step toward improving N use efficiency, and estimation of seed yield under different N regimes can be used as an indicator of global N use efficiency (Bouchet et al., 2016).

It has been hypothesized that rapeseed yield might be improved by optimizing density and N rates by alteration of canopy architecture, intercepted light and photosynthesis. Therefore, objectives of present field experiments were: (1) to examine the effects of different plant densities and N rates on yield, lodging resistance, N use efficiency in rapeseed and (2) to explore the relationship between lodging resistance and morphological and yield traits.

## Materials and methods

### Site characteristics and field trial management

The experiment was laid out in a split-plot design with three replications. The previous crop for the plots sown at both growing seasons was rice. The total experimental area was 840 m^2^, and the size of each main plot was 2 × 40 m (width × length) containing four sub-plots. Three plant densities (LD, 10 plants m^−2^; MD, 30 plants m^−2^; and HD, 60 plants m^−2^) were assigned to the main plots and four N rates (N_0_, 0 kg N ha^−1^; N_60_, 60 kg N ha^−1^; N_120_, 120 kg N ha^−1^; and N_180_, 180 kg N ha^−1^) to the sub-plots. The treatments were replicated three times to give a total of 36 experimental units. Seeds of Huayouza 9, a rapeseed hybrid widely cultivated in central China, were manually sown in rows at a depth of 2–3 cm, on September 26, 2014 and October 4, 2015. Distance between the rows was maintained at 30–35 cm. Distance within each row was adjusted by dense seeding and then thinned by hand to attain desired density rates when seedlings had reached at 4–5 leaves stage. Nitrogen was broadcasted as urea fertilizer (46% N), in three split doses: 50% before sowing, 30% during the over-wintering period, and 20% during the bud development period. No herbicides were applied to the field experiment and weeds were controlled by hand weeding. To control aphid, 50% Imidacloprid, 40–60 g wettable powder was mixed in 40–50 L water and was sprayed in 667 m^2^ when aphid strain rate was 8%. To control cabbage worm, 120 ml, 48% chlorpyrifos EC 100–120 ml was sprayed in 667 m^2^, when larva 2–3 cm. Fungicide 40% dimethachlon 0.1–0.2 kg/40–50 L water was sprayed at the beginning of flowering to control *Sclerotinia sclerotiorum*.

Soil samples were collected from the research field at a depth of 0–15 cm before sowing, air-dried, sieved, and analyzed in the laboratory using standard techniques. Total N was determined by the Kjeldahl method (Bremner, 1965). The available N content was determined using 1 M potassium chloride (KCl) extraction, followed by colorimetric analysis (Keeney, 1982). Available P was determined by the Olsen method according to Black (1965), organic content by the titrimetric method (Walkley and Black, 1934), and available K with a flame photometer (Knudsen et al., 1982). Physicochemical properties of the soil are presented in Table 1.

**Table 1:** **Physico-chemical properties of the soil before the study conducted in 2014/15 and 2015/16**.

**Soil samples collected**	**Texture**	**pH**	**Organic matter (g kg^−1^)**	**Available N (mg kg^−1^)**	**Available P (mg kg^−1^)**	**Available K (mg kg^−1^)**	**Total N (%)**	**Total P (%)**	**Total K (%)**
2014/15	Silt clay loam	6.45	8.01	64.5	9.81	148	0.07	0.03	1.11
2015/16	Silt clay loam	6.12	8.40	72.4	10.30	145	0.09	0.04	1.40

### Meteorological conditions

Meteorological data were collected for both growing seasons (2014/15 and 2015/16) from local weather station and they are presented in Table 2. The total hours of sunshine, accumulative temperature, and rainfall in 2014/2015 were slightly higher than those in the 2015/2016 growing season.

**Table 2:** **Meteorological conditions during the study conducted for two consecutive winter rapeseed-growing seasons**.

**Growing season**	**Meteorological condition**	**Growth stages**				
		**Seedling**	**Wintering**	**Flowering**	**Pod filling**	**Total growth**
2014/15	Total precipitation (mm)	252.1	89.7	88.3	203.5	635.2
	Effective accumulated temperature (°C)	1,034.6	24.6	240.9	565.9	1,833.6
	Solar radiation (MJ m^−2^)	1,656.3	206.5	362.2	650.5	2,860.5
2015/16	Total precipitation (mm)	242.2	66.7	125.2	168.4	605.4
	Effective accumulated temperature (°C)	958.2	80.6	271.5	522.7	1,825.4
	Solar radiation (MJ m^−2^)	1,102.4	190.1	341.4	525.5	2,065.3

### Seed yield and yield-related traits

On May 15, 2015 and May 18, 2016, at maturity 10 plants from each plot were randomly selected and roots were carefully dug out from 30 cm depth of soil by using a spade to determine plant height, root weight, root neck diameter, number of pods per plant, number of seeds per pod, and the weight of 1,000 seeds. Plant height was measured from the base to the highest bud. The roots were cut from the shoot, washed to remove soil, weighed, and root neck diameter was measured using Vernier calipers. The shoots and roots were oven-dried at 72°C until constant weight and their biomass was measured. The remaining plants in each plot were harvested by hand to measure seed yield. Pod area index was calculated from 10 randomly selected plants from each plot as follows: Sa = πd × (h_1_ + 1/3 × h_2_), where h_1_ is 0.8 of the pod length, h_2_ is 0.2 of the pod length, and d is pod width.

### Lodging resistance, lodging resistance index (LRI), and lodging-related morphological traits

A lodging tester (Hangzhou TOP Instrument Co., Hangzhou, China) was used to measure lodging resistance. The tester was set perpendicularly in the middle of the second basal internode, and the strength (kg cm) needed to break stem internode was measured. The LRI was computed as follows (Peng et al., 2014): LRI = (stem height at the center of gravity × shoot fresh weight/breaking strength of the second basal internode) × 100.

### Nitrogen use efficiency, net photosynthetic rate, and light interception ratio (LIR)

Plant N concentration was determined by the micro-Kjeldahl method (Ozer, 2003). Apparent recovery nitrogen use efficiency (ARNUE) was evaluated (Li et al., 2014):

$$\text{ARNUE}=[({\text{NU}}_{\text{fi}}-{\text{NU}}_{\text{f}0})/{\text{N}}_{\text{f}}]\times 100,$$

Where, NU_fi_: N uptake of fertilized plants (kg ha^−1^), NU_f0_: N uptake of unfertilized plants (kg ha^−1^), N_f_: N fertilizer applied (kg ha^−1^).

Photosynthetic rate of the topmost, the middle, and the lowest leaf of intact plants was measured from 09:30 to 11:00 using Li-6400 (Li-COR Inc., Lincoln, NE, USA) under a light intensity of 1,500 mol m^−2^ s^−2^. Measurements for leaf photosynthesis were started at bud stage (BBCH 20) and performed with 14 days intervals until the onset of flowering (BBCH 60). Light interception refers to the amount of solar radiation intercepted by foliage and other green tissues. Light interception was measured using a SunScan Canopy Analysis System (Delta-T Devices Ltd., UK), during the growing season until the onset of flowering (BBCH 20–60), between 11:00 and 15:00. To measure intercepted light, 1 m probe was set perpendicular to soil surface and two measurements were recorded above the canopy and two measurements below the canopy, with a third below-canopy measurement in low-density plots. Light interception was calculated as (Liu et al., 2012):

$$\begin{array}{l} \text{LIR }(\%)\;=\;[1\;-\;(\text{Average PAR below canopy/PAR above}\\ \text{ canopy})]\;\times \;100. \end{array}$$

### Statistical analysis

Analysis of variance (ANOVA) in conjunction with Duncan's multiple range test was applied to identify significant differences between treatment levels and combinations of treatments at *p* < 0.05. All analyses were carried out using SAS 8.1 (SAS Corp., Cary, NC, USA), and graphs were constructed using Microsoft Excel 2010 (Microsoft Corp., Redmond, WA, USA).

## Results

### Seed yield and yield-related traits

The seed yield increased significantly with the increase in plant density and nitrogen in both growing seasons, reaching maximum levels at HD and N_180_ (Table 3). The weight of 1,000 seeds increased significantly with the application of nitrogen, reaching the maximum at N_180_, whereas plant density and D × N did not affect significantly the 1,000-seed weight. Plant density had no significant effect on the effective number of pods per plant, whereas N had a significant positive effect, with the maximum number of pods per plant observed at N_120_ and minimum at N_0_. Under LD, the number of branches increased with the increasing N, reaching maximum at N_180_, however at higher densities, branches numbers increased up to N_120_, and declined at N_180._ The pod area index increased significantly with increasing plant density and N rate, reaching a peak at HD and N_180_ in both growing seasons (Table 3).

**Table 3:** **Effect of plant densities and N rates on seed yield, 1,000-seed weight, number of pods per plant, number of branches, and pod area index in the growing season of 2014/15 (I) and 2015/16 (II)**.

		**Seed yield (kg ha^−1^)**		**1,000 grains weight (g)**		**Effective pods (no. plant^−1^)**		**Effective branches (no. plant^−1^)**		**Pod area index**	
		**I**	**II**	**I**	**II**	**I**	**II**	**I**	**II**	**I**	**II**
**TREATMENTS**											
Density	LD	2,893	3,095	3.04	3.07	185.6	206.3	6.51	6.09	4.05	3.35
	MD	3,216	3,276	2.98	3.08	195.5	208.3	6.11	5.50	4.46	4.23
	HD	3,794	3,708	2.84	2.78	180.1	199.3	6.74	6.42	6.36	5.59
Nitrogen	0	2,422	1,712	2.55B	2.72C	69.3B	86.9B	2.64	2.33	4.31	3.78
	60	3,478	3,677	2.83B	2.94BC	220.6A	240.1A	7.03	6.78	4.58	4.09
	120	3,571	3,927	3.21A	3.07AB	245.2A	243.9A	8.05	7.45	5.29	4.53
	180	3,732	4,122	3.21A	3.17A	213.1A	247.6A	8.09	7.45	5.64	5.15
**INTERACTIONS**											
LD	0	1,464e	1,528d	2.60	2.73	50.2	62.5	2.30f	2.00e	3.46h	3.09e
	60	3,309c	3,374c	2.97	2.93	217.6	248.0	6.43d	6.00d	3.98fgh	3.24e
	120	3,396bc	3,701bc	3.30	3.37	254.0	260.4	8.30ab	7.67abc	4.24fg	3.49e
	180	3,402bc	3,777bc	3.27	3.23	220.7	254.3	9.00a	8.67a	4.51ef	3.56e
MD	0	2,563d	1,695d	2.73	2.93	91.3	117.0	2.10f	2.00e	3.62gh	3.43e
	60	33,310c	3,426c	2.83	3.10	233.0	236.0	6.90cd	6.33d	3.83gh	3.63e
	120	3,386bc	3,926bc	3.17	2.97	236.8	240.7	7.77bc	7.00bcd	5.05de	4.46d
	180	3,603abc	4,057ab	3.20	3.31	220.7	239.4	7.67bc	6.67cd	5.34cd	5.38bc
HD	0	3,239c	1,913d	2.31	2.50	66.3	81.2	3.53e	3.00e	5.86c	4.81cd
	60	3,814abc	4,232ab	2.70	2.80	211.3	236.3	7.77bc	8.00ab	5.93bc	5.39bc
	120	3,932ab	4,155ab	3.17	2.87	244.7	230.6	8.07ab	7.67abc	6.58ab	5.65b
	180	4,191a	4,532a	3.17	2.87	198.0	249.0	7.60bc	7.00bcd	7.08a	6.52a
Y		ns		ns		ns		ns		ns	
D		^**^		ns		ns		^*^		^**^	
N		^***^		^***^		^**^		^***^		^**^	
Y × D		ns		ns		ns		ns		ns	
Y × N		ns		ns		^*^		ns		ns	
D × N		^*^		ns		ns		^*^		^*^	
D × N × Y		ns		ns		ns		^*^		ns	

ns, non-significant;

* significant at p < 0.05;

** significant at p < 0.01; and

*** *significant at p < 0.001. For significant interaction, differences among treatments are indicated by different lowercase letters; for non-significant interaction, differences within treatment are indicated by uppercase letters*.

### Lodging resistance, LRI, and lodging-related traits

Lodging resistance and LRI were affected significantly by plant density and nitrogen. Lodging resistance was increased by increasing the density from 10 to 30, but it decreased by further increase of density to 60 plants m^−2^ (Table 4). N application increased significantly the lodging resistance, peaking at 120 kg ha^−1^. D × N affected the lodging resistance, with the maximum resistance observed at MD at 120 kg N ha^−1^ and the minimum at LD without N. The LRI at LD and MD decreased and at HD increased with increasing the N. The maximum value of LRI recorded at HD was at 180 kg N ha^−1^ and the minimum at MD at 120 kg N ha^−1^ in both growing seasons.

**Table 4:** **Effect of plant densities and N rates on lodging resistance, lodging resistance index (LRI), photosynthetic rate (***P***_**n**_), light interception ratio (LIR), and apparent recovery nitrogen use efficiency in the growing season of 2014/15 (I) and 2015/16 (II)**.

		**LR (kg cm)**		**LRI (%)**		***P_n_* (μmol m^−2^ sec^−1^)**		**LIR (%)**		**N use efficiency (%)**	
		**I**	**II**	**I**	**II**	**I**	**II**	**I**	**II**	**I**	**II**
**TREATMENTS**											
Density	LD	4.12	4.03	2.01	1.56	7.31	7.83	64.48	62.75	15.83B	15.03C
	MD	7.30	5.60	1.99	1.23	9.40	6.47	72.05	70.13	21.83A	22.53A
	HD	4.65	3.35	4.16	1.79	8.26	6.47	72.45	70.80	19.40A	17.97B
Nitrogen	0	5.31	3.91	3.29	1.85	4.30	4.00	66.07	66.03		
	60	5.46	4.85	3.10	1.54	8.45	7.14	68.30	66.63	21.57A	20.57A
	120	5.45	4.41	1.95	1.48	10.11	7.75	71.87	66.87	20.13A	19.77A
	180	5.19	4.12	2.55	1.24	10.43	8.79	72.40	72.03	15.37B	15.20B
**INTERACTIONS**											
LD	0	3.47c	1.77c	3.55abc	2.15a	4.27e	3.91g	60.6e	60.6d	–	–
	60	3.77c	4.40b	2.33bcde	1.44c	7.10d	6.91cde	62.4e	62.6bcd	19.1	16.4
	120	3.73c	4.97ab	0.49e	1.82abc	8.71bcd	9.37ab	66.9bcde	63.5bcd	16.4	17.6
	180	5.50bc	4.97ab	1.66cde	0.83de	9.15bcd	11.12a	68.0bcde	64.3bcd	12.0	11.1
MD	0	5.91abc	5.53a	2.98abcde	2.13ab	4.39e	3.92g	65.4bcde	62.2cd	–	–
	60	8.30a	5.63a	3.79abc	1.51bc	9.85abc	8.83bc	68.4bcd	64.5bcd	23.5	25.2
	120	8.59a	5.73a	0.47e	0.56e	11.09ab	6.12def	73.4abcd	74.4abc	24.1	22.5
	180	6.38ab	5.50a	0.73de	0.72de	12.28a	7.00cde	81.0a	79.4a	17.9	19.9
HD	0	6.56ab	4.43b	3.34abcd	1.26cd	4.23e	4.17fg	72.2abc	75.3ab	–	–
	60	4.32bc	4.53b	3.18abcd	1.66abc	8.41cd	5.69efg	74.1abc	72.8abcd	22.1	20.1
	120	4.03bc	2.53c	4.88ab	2.07ab	10.52abc	7.77bcd	75.3ab	62.7bcd	19.9	19.2
	180	3.70c	1.90c	5.25a	2.18a	9.86abc	8.26bc	68.2bcd	72.4abcd	16.2	14.6
Y		ns		ns		^*^		ns		ns	
D		^**^		^**^		^*^		^*^		^**^	
N		^***^		^*^		^**^		^**^		^**^	
Y × D		ns		ns		ns		ns		ns	
Y × N		ns		ns		ns		ns		ns	
D × N		^*^		^**^		^*^		^*^		ns	
D × N × Y		^*^		ns		ns		ns		ns	

ns, non-significant;

* significant at p < 0.05;

** significant at p < 0.01; and

*** *significant at p < 0.001. For significant interaction, differences among treatments are indicated by different lowercase letters; for non-significant interaction, differences within treatment are indicated by uppercase letters*.

**Figure 1:**
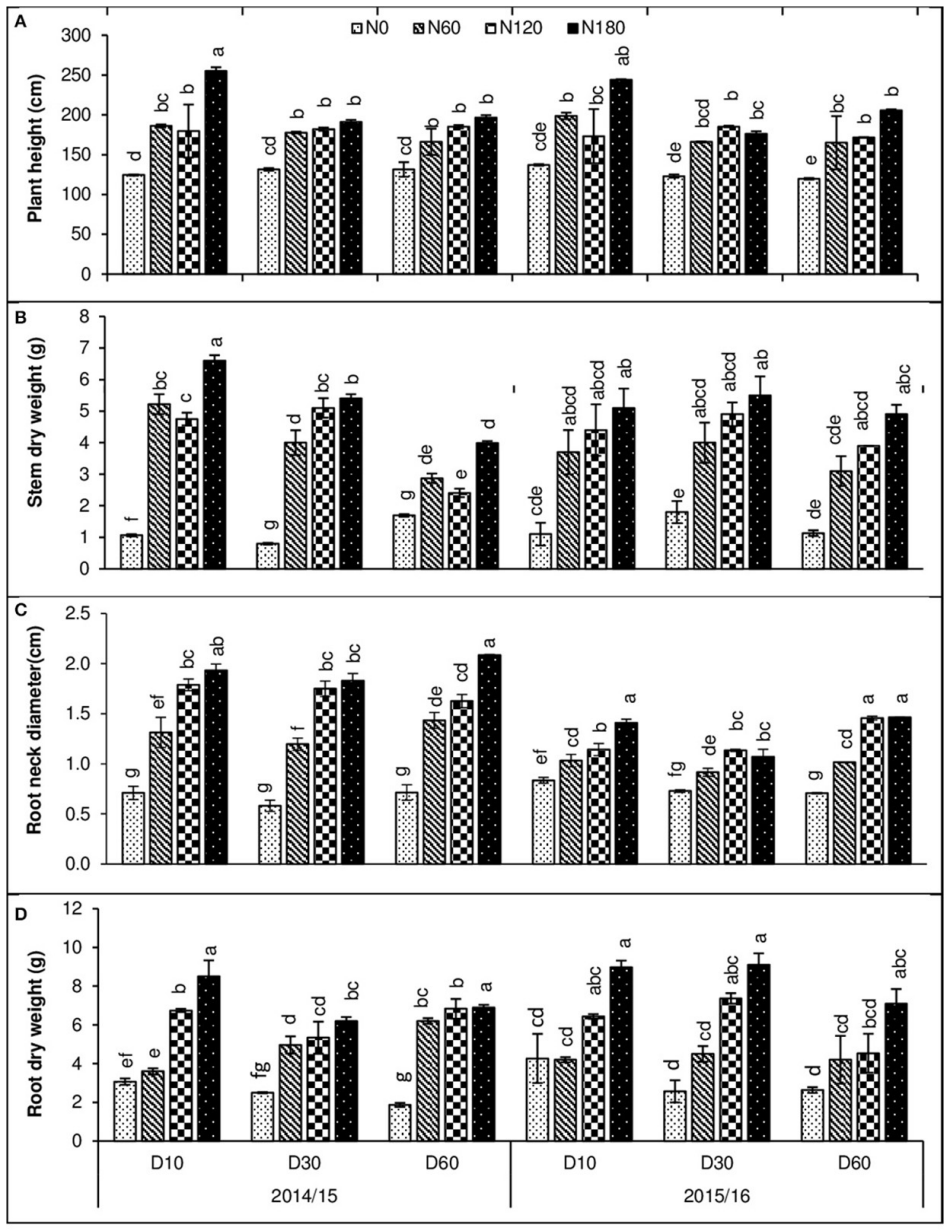
**Effect of plant densities (10, 30, and 60 plants m^**−2**^) and N rates (0, 60, 120, and 180 kg ha^**−1**^) on rapeseed, (A)** plant height, **(B)** shoot dry weight, **(C)** root neck diameter, and **(D)** root dry weight in two consecutive growing seasons (2014/15 and 2015/16). Error bars indicate standard errors. Different letters indicate significant differences at *p* < 0.05.

The effects of density and N rates on some lodging-related morphological traits were studied (Figure 1). Plant height increased by increasing N levels, whereas it decreased at high plant density; the maximum height was observed at LD with 180 kg N and the minimum at HD without N in both growing seasons (Figure 1A). The shoot dry weight was increased with increasing nitrogen rates, reaching a peak at 180 kg ha^−1^ for all three plant densities. Shoot dry weight was more at LD and MD which was reduced at HD (Figure 1B). The root neck diameter and root dry weights were increased by N rate in both growing seasons, reaching a peak at 180 kg N ha^−1^ for all three plant densities.

### Net photosynthetic rate, light interception, and nitrogen use efficiency

Density affected significantly the net photosynthetic rate and LIR; both reached a peak at 30 plants m^−2^ and then declined as density increased to 60 plants m^−2^ (Table 4). The net photosynthetic rate and LIR at LD and MD showed a significant increase as N rate increased from 0 to 180 kg ha^−1^. At HD, LIR was increased by increasing N rate from 0 to 120 kg ha^−1^, further increase of N rate to 180 kg ha^−1^, decreased the LIR. The D × N interaction significantly affected the net photosynthesis and LIR; the minimum values of both were observed at LD without N. Within the same density rate, NUE was declined by increasing the N rate, in contrast to which by increasing density rate from 10 to 30 plants m^−2^ NUE was increased, and at 60 plants m^−2^ was also higher than 10 plants m^−2^.

### Correlation of lodging resistance with yield and other traits

Lodging resistance was significantly and positively correlated with seed yield, shoot and root dry weight, root neck diameter, and pods per plant but negatively related with plant height and lodging resistance index. Seed yield was positively correlated with plant height, root neck diameter, and root dry weight (Table 5).

**Table 5:** **Correlation coefficient (***r***) among plant height, shoot dry weight, root dry weight, root neck diameter (ND), 1,000-seed yield, number of pods per plant, lodging resistance (LR), and lodging resistance index (LRI) in rapeseed**.

**Traits**	**Plant height**	**Shoot DW**	**Root DW**	**Root ND**	**Seed yield**	**Pods plant^−1^**	**LR**	**LRI**
Plant height	–	–	–	–	–	–	–	–
Shoot DW	0.31^**^	–	–	–	–	–	–	–
Root DW	0.29^*^	0.80^***^	–	–	–	–	–	–
Root ND	0.41^***^	0.44^***^	0.37^**^	–	–	–	–	–
Seed yield	0.43^***^	0.15ns	0.65^***^	0.46^***^	–	–	–	–
Pods plant^−1^	0.59^***^	0.63^***^	0.61^***^	0.57^***^	0.38^***^	–	–	–
LR	0.03ns	0.29^*^	0.30^**^	0.44^***^	0.49^***^	0.39^***^	–	–
LRI	0.31^*^	0.03ns	0.02ns	0.04ns	−0.20ns	0.15ns	−0.29^*^	–

ns, non-significant;

* significant at p < 0.05;

** significant at p < 0.01; and

*** *significant at p < 0.001*.

## Discussion

Plant density is an important factor for establishing a uniform crop stand to ensure high yield. Moreover, N is probably the most important nutrient in rapeseed production because its deficiency results in yield reduction (Jackson, 2000; Begdelo et al., 2011). In the present study, seed yield increased with increasing plant density and N rate, reaching a peak at high density (HD) and 180 kg N ha^−1^. Ahmadi and Bahrani (2009) reported the highest yield for rapeseed at N concentration of 225 kg ha^−1^. This linear relationship between seed yield and N rate could be attributed to higher number of pods per plant, seeds per pods, and seed-carrying pods (Ghanbari-Malidarreh, 2010; Imran et al., 2014).

In present study, N use efficiency was improved with increasing the density rate from 10 plants m^−2^ to 30 and 60 plants m^−2^. Li et al. (2014) compared two plant density levels and reported that high density (45 plants m^−2^) in rapeseed improved N use efficiency compared to low density of 15 plants m^−2^. The present study showed that N use efficiency was less at 180 kg N ha^−1^ as compared to lower applied N rates. It indicated that rapeseed plants were unable to uptake N at high N rate because the supply of N in excess of plant requirement and possibility exists for the loss of N by leaching and denitrification. Plant N concentration and N use efficiency mostly decline at the maturity stage due to the greater dry matter accumulation rate than N accumulation rate (Chamorro et al., 2002). Significant proportion of leaf N was not mobilized before abscission of leaves during flowering and pod filling resulting in low N recovery by plant at maturity (Malagoli et al., 2005).

In the present study, the number of branches was greater at low plant density (LD) and the highest applied N rate (N_180_). Tunçtürk and Çiftçi (2007) reported a positive correlation between seed yield and the number of branches. However, in the present study, the seed yield was greater at higher plant density despite the lesser number of branches and pods per plant, which might be correlated with increased lodging resistance due to reduced plant height and greater number of plants with uniform canopy at higher densities. Li et al. (2014) reported that increasing planting density from 15 to 45 plants m^−2^ significantly decreases plant height, branch number, and effective pod number per plant and thereby makes the canopy uniform. Less competition for assimilates at low plant density stimulates the growth of the apical and lateral meristems, resulting in higher number of branches and uneven, delayed maturation (Inamullah et al., 2013). Our results showed that the number of branches increased with N application; however, the number of branches decreased with increasing plant density at N_120_ and N_180_, probably because at high N rates, plant growth is more vigorous and the increase in plant density negatively affects the number of branches (Ozer, 2003). Low planting density promotes plant growth, leading to taller plants with increased number of branches and pods per plants. Such plants require more nitrogen as compared to plants at higher densities (Šidlauskas and Tarakanovas, 2004). N application also increased the number of pods per plant in both growing seasons, which could be attributed to a higher number of pod-bearing branches (Ozer, 2003).

Lodging is a serious obstacle for rapeseed production particularly in rapeseed growing areas of Yangtze River basin, receiving high rainfall. Resistance to lodging depends on morphological, physiological, and biochemical traits and is related to stem strength, plant height, wall thickness, lignin content, and center of gravity (Kong et al., 2013; Chen et al., 2014; Ookawa et al., 2014). Lodging resistance (LR) and lodging resistance index (LRI) are closely associated with the actual lodging score at the field level (Islam et al., 2007). In the present study, at medium density (30 plants m^−2^), LR increased with N application, reaching a peak at 120 kg ha^−1^, but decreased by further increase to 180 kg ha^−1^, whereas at high density rate (60 plants m^−2^), LR was decreased with increasing N rate. Zhang et al. (2014) reported an increase in lodging due to N application, which was associated with enhanced plant growth and height. Similarly, LR increased with increasing the plant density up to 30 plants m^−2^, which was then followed by a decrease at the highest studied density (60 plants m^−2^). Previous studies showed that the risk of lodging increases under conditions of relatively high plant density because of the longer basal internode, higher center of gravity, and decreased shoot diameter of plants (Mobasser et al., 2008, 2009; Xiao et al., 2015). In the present study, LR showed positive correlation with seed yield and dry biomass, whereas the correlation with plant height was non-significant. The maximum LR was recorded at MD using 120 kg N ha^−1^ and the minimum plant height was at HD without N. Therefore, minimum plant height does not warrant high lodging resistance and is not necessarily the most important factor determining lodging resistance (Ookawa and Ishihara, 1992). Plant height could be increased without affecting the lodging if the breaking resistance and dry weight per unit length are also increased (Islam et al., 2007). In the present study, the shoot and root dry weight and the root neck diameter were increased with increasing plant density and nitrogen rate, reaching a peak at highest used N (180 kg ha^−1^), which might be another possible reason for increased lodging resistance in plants. Root traits are also valuable for determining lodging, since resistant genotypes usually have a well-developed root system that increases the anchorage strength of the plant.

LRI is an important parameter for estimating lodging. This index is affected by a number of plant traits such as plant height, shoot weight, stem thickness, and breaking strength (Yang et al., 2000; Mobasser et al., 2009; Peng et al., 2014). Our results showed that the minimum LRI was found at medium density (30 plants m^−2^) along with 120 kg N ha^−1^. Increasing density (60 plants m^−2^) and N (180 kg ha^−1^) then increased the LRI and thus weaker lodging resistance, which subsequently causes lodging and yield losses. A negative relationship between LR and plant height indicated that increase in plant height reduces the lodging resistance of rapeseed. Increase in breaking resistance of lower internodes and dry biomass per unit length are the main morphological traits responsible for reduction in the lodging index and thus the induction of lodging resistance (Islam et al., 2007).

In the present study, light interception ratio (LIR) and photosynthetic rate was at peak at highest N (180 kg ha^−1^) and medium plant density (MD), whereas decreased at lowest (10 plants m^−2^) and highest density (60 plants m^−2^) rates. It is known that LIR is related to biomass production—relatively high plant densities hasten canopy interception and increase yield (Portes and Melo, 2014), whereas solar radiation is not fully intercepted at relatively low plant densities (Atwell et al., 1999). Concurrently, relatively high plant densities do not allow light to reach lower shoot sections, triggering shoot elongation. However, if the quantity and quality of light are not disturbed, the shoot elongation rate is not affected by plant density (Holmes and Smith, 1977). Plant density affects photosynthesis by influencing the radiation interception and the structure of the canopy (Ma et al., 2014). Since lodging may reduce the photosynthetic capacity of the canopy, another target is needed to improve lodging resistance. However, relatively high plant densities are negatively correlated with photosynthetically active radiation (Tetio-Kagho and Gardner, 1988). These results indicated that rapeseed yield might be improved by selecting the density and nitrogen rates at which plants have optimum height, shorter basal internodes, and uniform canopy with high light interception—traits that are associated with higher lodging resistance (Zhang et al., 2014).

## Conclusion

Increasing plant density and nitrogen rate to the maximum studied rates significantly increased the seed yield. Lodging resistance was increased by increasing plant density from 10 to 30 plants m^−2^ and N from 0 to 120 kg ha^−1^ and decreased by further increase in density and N rate. The net photosynthetic rate and light interception showed same trend—reached a peak at 30 plants m^−2^ at 180 kg N ha^−1^. N use efficiency was increased at higher plant density and decrease by increasing N rates. It might be concluded from these results that the density rate of 30 plants m^−2^ and 180 kg N ha^−1^ is appropriate to attain high yield of rapeseed, N use efficiency and lodging resistance in winter rapeseed.

## Author contributions

GZ designed and supervised the research project. SK performed the experiments and collected the data. SU helped in data collection. SK and SA analyzed the data and wrote the manuscript. JK and SF revised and edited the manuscript and also provided advice on the experiment. GZ read and approved the final manuscript.

### Conflict of interest statement

The authors declare that the research was conducted in the absence of any commercial or financial relationships that could be construed as a potential conflict of interest.
